# Population Dynamics and Reintroduction Strategies for the Alpine Marmot in Romania

**DOI:** 10.3390/ani15172496

**Published:** 2025-08-25

**Authors:** Alexandru Gridan, George Sîrbu, Iulia Baciu, Georgeta Ionescu, Ovidiu Ionescu, Darius Hardalau

**Affiliations:** 1 Department of Wildlife, National Institute for Research and Development in Forestry “Marin Drăcea”, 500040 Brasov, Romania; gridanalex@gmail.com (A.G.); sirbugeorgeeugen@gmail.com (G.S.); iuliaa.baciu@yahoo.com (I.B.); titi@icaswildlife.ro (G.I.); 2 Silviculture Department, Faculty of Silviculture and Forest Engineering, Transilvania University of Brasov, 500036 Brasov, Romania; darius.hardalau@unitbv.ro

**Keywords:** alpine marmot, reintroduction, population growth, wildlife conservation

## Abstract

The alpine marmot (*Marmota marmota*) once vanished from Romania’s Carpathian Mountains, but a reintroduction initiative launched in 1973 has since fostered population recovery. This study evaluates the long-term success of those efforts and explores the feasibility of further reintroductions. Population data from 2004 to 2025 across the Rodna, Retezat, and Făgăraș Mountains show a steady growth trend, culminating in a national population of approximately 815 individuals by 2025. Habitat quality was assessed using a Habitat Suitability Index, identifying robust colonies that could serve as donor populations. Additionally, an Ecological Diagnostic Key was developed to evaluate 27 potential reintroduction sites in the Țarcu, Ciucaș, and Bucegi Mountains. Findings reveal that 61 ha are suitable for reintroduction, with 40.5 ha rated as highly suitable. The study proposes a phased reintroduction strategy, combining individuals from established Romanian colonies with genetically diverse founders from Western Europe. It emphasizes soft-release protocols and calls for mitigation of anthropogenic pressures. This research supports a broader conservation framework that integrates habitat evaluation, demographic stability, and genetic health to enhance the alpine marmot’s persistence and expansion in Romania.

## Simple Summary

Through reintroduction efforts in 1973, the alpine marmot was successfully reintroduced to Romania. This study aims to evaluate the current status of the alpine marmot population and assess the potential for reintroducing the species into three additional mountain ranges. Over the past two decades, the population has shown a positive growth trend, reaching approximately 815 individuals by 2025. To determine whether the populations in the Retezat, Rodna, and Făgăraș Mountains are strong and stable enough to serve as source populations, a Habitat Suitability Index was developed to identify suitable relocation areas. To evaluate potential reintroduction sites, 27 locations in the Țarcu, Ciucaș, and Bucegi Mountains were assessed using an Ecological Diagnostic Key, revealing 40.5 hectares of highly suitable habitat. A phased reintroduction strategy is recommended, incorporating soft-release protocols and using marmots from both Romanian and external populations to ensure genetic stability.

## 1. Introduction

The genus Marmota includes 15 species of ground squirrels distributed across Europe, Asia, and North America, inhabiting ecosystems ranging from steppe environments to the highest alpine altitudes [1,2,3]. The alpine marmot (*Marmota marmota* L.) is a key species of high-altitude habitats in Europe, with populations found in the Pyrenees, Alps, and Carpathian Mountains [4]. Alpine marmots are highly social mammals that live in colonies or extended family groups, exhibiting complex behaviors such as cooperative vigilance, both inter- and intraspecific [5,6]. Like other hibernating mammals [7,8], marmots undergo seasonal dormancy, but uniquely, they hibernate communally in deep burrows to conserve heat and energy [9,10]. Adapted to survive in some of the harshest alpine environments, characterized by long winters and a short vegetation season, the alpine marmot exemplifies resilience in cold-climate ecosystems [11]. It plays a vital ecological role by modifying soil structure through extensive burrowing [12], contributing to nutrient cycling [13,14,15], and serving as prey for apex predators [16] such as the golden eagle (*Aquila chrysaetos*) [17]. Due to its sensitivity to anthropogenic disturbances, including grazing pressure and habitat degradation [18,19], the alpine marmot is considered a valuable bioindicator of ecosystem integrity. The alpine marmot remains the most widespread alpine rodent, being found in the alpine regions of France [20,21], Italy [22], Austria [23], and other European countries. Successful translocations have created robust colonies in the Pyrenees [15,20,24], whose demographic profiles mirror those of long-established Alpine colonies [15].

The historical presence of the alpine marmot in Romania has long been a topic of debate due to scarce archeological evidence and the absence of a native Romanian term for the species [25]. Sporadic mentions in historical documents and place names, such as those found in the Făgăraș, Rodna, and Retezat Mountains, suggest the species may have once inhabited the Romanian Carpathians but disappeared before the 20th century [26]. In the early 1970s, efforts to reintroduce alpine marmots were initiated to reestablish their ecological role in high-altitude ecosystems [27]. In 1973, 50 marmots captured from the French Vanoise National Park and the Austrian Alps near Salzburg were released into three locations: the Rodna Mountains (12 individuals), the Retezat Mountains (17 individuals), and the Făgăraș Mountains (21 individuals). These sites were chosen because they fit the species’ natural conditions and Romanian sources had them associated with the species. The release was a part of a coordinated effort led by Romanian authorities, wildlife researchers, and hunters, aiming to restore a species that had disappeared from the country by the end of the 19th century [25].

The success of this action laid the foundation for future population growth and the species’ establishment in the Carpathians alpine areas. Despite initial challenges, including abandoned artificial burrows, the marmots adapted well, gradually expanding their ranges within glacial cirques and alpine meadows [25]. Marmots primarily feed on alpine plant species such as *Carex curvula*, *Juncus trifidus*, *Agrostis rupestris*, *Festuca airoides*, *Nardus stricta*, *Sesleria tenuifolia*, and *Poa alpina*, as well as fruits from shrubs like *Vaccinium myrtillus* and *Vaccinium vitis-idaea* [25,28,29]. The availability of these plants serves as a limiting factor for marmot survival. The species has found suitable ecological niches in scree areas and coexists peacefully, through mutualism, with native species such as the chamois (*Rupicapra rupicapra*), with which it may share interspecific vigilance through alarm whistling in response to potential threats [11,30]. The grazing pressure is high in alpine meadows across the Romanian Carpathians and, together with other influencing factors such as heat and tourism, marmots forage during the early morning or late evening hour, periods that overlap with peak activity times of carnivores and other predators. Furthermore, the population density of large carnivores such as the brown bear (*Ursus arctos*), gray wolf (*Canis lupus*), and European lynx (*Lynx lynx*) in Romania is significantly higher than in other European countries, which may limit marmot population growth compared to other alpine regions [31]. Nevertheless, the steady population increase observed since reintroduction remains a strong example of the Allee effect, where group-living and social cooperation enhance survival and reproductive success in small or reintroduced populations [32,33].

Given the success of the alpine marmot reintroduction and the species’ ability to survive and thrive over the past half-century [25], it is notable that marmots are still absent from many alpine regions of Romania. At the same time, not all alpine meadows are currently used for grazing, and traditional grazing practices appear to be gradually declining [34]. Therefore, this study aims to evaluate whether current alpine marmot (*Marmota marmota*) populations in Romania are stable and exhibiting sustained growth, and to assess the feasibility of relocating individuals either from existing Romanian populations or from other European populations to avoid inbreeding and establish new viable colonies. To address these objectives, the study first analyzes population trends over the past two decades in the three reintroduction regions (Făgăraș, Retezat, and Rodna). Second, it evaluates the habitat quality and natural resources of currently occupied territories to determine whether donor colonies could support translocation. Finally, the study assesses habitat suitability in three additional alpine regions (Țarcu, Ciucaș, and Bucegi Mountains) using a standardized ecological diagnostic index to identify candidate sites for future reintroductions.

## 2. Materials and Methods

### 2.1. Study Area

This study was conducted across six core alpine regions in Romania where reintroduced populations of alpine marmot (*Marmota marmota*) are already established: the Retezat, Făgăraș, and Rodna Mountains; or in areas where the marmot can be reintroduced: Ciucaș, Bucegi, and Țarcu Mountains. The first three are the original sites of reintroduction in 1973 and continue to support the only marmot populations in Romania.

The Retezat Mountains, situated in the Southern Carpathians, encompass the alpine and subalpine zones of Retezat National Park. Elevations range from approximately 1300 m to over 2500 m. The landscape is shaped by glacial valleys, a dense network of glacial lakes, and a mosaic of alpine vegetation. The climate is montane to alpine, with average annual temperatures between 4 and 6 °C and persistent snow cover lasting up to 200 days per year. The Făgăraș Mountains are also part of the Southern Carpathians, span altitudes between 1400 and 2150 m, and are characterized by steep, north-facing slopes, glacial valleys, and rugged terrain. Hydrologically, the area includes numerous streams and tributaries feeding into the Olt River. The climate is alpine, with long winters, cool summers, and annual precipitation exceeding 1000 mm. Being located in the Eastern Carpathians, the Rodna Mountains rise between 1600 and 2300 m in elevation. The region features a mix of alpine meadows, grasslands, and subalpine shrublands, with less pronounced glacial landforms than the Southern Carpathians. The climate is typically alpine, marked by cool summers, moderate precipitation, and a long period of snow cover.

The Bucegi Mountains, located in the Southern Carpathians, are part of Bucegi Natural Park and span elevations from approximately 1600 m to 2505 m at Omu Peak. The area features extensive alpine meadows, subalpine pastures, and rocky ridges with predominantly southern and eastern exposures. The climate is alpine–continental, characterized by cold winters, cool summers, and annual precipitation exceeding 1000 mm. Human impact varies a lot: some areas experience high tourism pressure, others are under strict protection. The presence of gramine-rich pastures and suitable soils for burrows enhances the potential for marmot recolonization. The Țarcu Mountains, part of the Western Carpathians, are among the most remote and least disturbed mountain ranges in Romania. Elevations range from 1200 m to 2190 m (Țarcu Peak). The landscape is defined by wide alpine meadows, glacial cirques, and subalpine grasslands. Human activity is minimal, with limited infrastructure and large areas of pristine vegetation and landscapes. The local climate is harsh, with long-lasting snow cover and moderate summer temperatures, providing stable ecological conditions for alpine species. The Ciucaș Mountains, situated at the intersection of the Eastern and Southern Carpathians, reach a maximum elevation of 1954 m at Ciucaș Peak. This massif is characterized by rounded ridges, interesting rock formations, and a mosaic of subalpine meadows, juniper thickets, and scattered alpine pastures. Although there are lower elevations registered, compared to other study areas, Ciucaș maintains a high-quality habitat, particularly in sectors with limited human presence. The climate is humid montane, with frequent cloud cover and consistent precipitation. Habitat fragmentation is more evident here, but certain zones remain moderately suitable for small-scale recolonization efforts.

### 2.2. Data Collection

To understand the dynamics of the alpine marmot in Romania, the population number size was collected from the Ministry of Environment, Waters and Forests for the past two decades (2004–2024). Estimates on alpine marmot populations were collected through direct observations conducted in early autumn (September), during the same period as the annual chamois population assessments, which allowed for coordinated field efforts, all of them according to Law 407/2006 [35] and Order 2847/2022 [36] instructions. Although this timing reflects the species pre-hibernation activity, it represents a practical strategy for data collection.

Observations were carried out using high-performance binoculars by camouflaged observers, with repeated counts over 1–2 days to ensure accurate identification and proper counting of all individuals (adults and juveniles) within each identified colony. Since all marmot surveys were conducted at the hunting ground level, and some areas were later included in the census of Romania’s National and Natural Park system, the data were adjusted and corrected to avoid presenting inaccurate information. More specifically, data were spatially aligned with the current boundaries of national and natural parks, and duplicates or overlapping entries were corrected using official administrative maps.

Data collection for the assessment of habitat suitability across the 34 alpine marmot colonies in the mountain ranges was conducted through direct observation by a team of four researchers. For each colony, the following variables were recorded either through field observation or with the aid of binoculars and GPS devices: altitude (in meters), exposition (recoded in eight classes), slope (in degrees), territory size (in ha), number of active colony entrances, number of abandoned entrances, and an assessment of anthropogenic pressure (three classes). While entrances were physically counted, the anthropogenic factor was evaluated on a scale from 1 to 3, based on the severity of sheep and cattle grazing, the presence of livestock guardian dogs, and the level of tourism pressure. Observations were carried out daily from 7:00 a.m. to 8:00 p.m. In the Retezat Mountains, data were collected in August; in the Făgăraș Mountains, observations occurred in May and September; and in the Rodna Mountains, fieldwork was conducted in September. The collected data were centralized (Table 1) and subsequently checked for outliers and potential recording errors.

In the case of the three mountain ranges identified for potential marmot reintroduction, the same research teams conducted assessments of possible future territories through direct observation. At these prospective colonization sites, the following variables were recorded: altitude (A), type of deposits (B), vegetation (C), and anthropogenic factor (D). For the type of deposits (B), the following categories were used: (i) no rocky outcrops, (ii) rocky outcrops in small patches, (iii) well-developed rocky outcrops in valleys with moderate slopes, and (iv) well-developed rocky outcrops in glacial cirques. For vegetation (C), four classes were defined: (i) no vegetation or tree-dominated vegetation, (ii) a mix primarily of mosses and lichens, with secondary presence of low-productivity grasses, (iii) a mix of medium-productivity grasses with alpine clovers, and (iv) a mix of high-productivity grasses with alpine clovers. The anthropogenic factor (D) was evaluated using the following scale: (i) intensive grazing pressure with excessive numbers of livestock guardian dogs, (ii) moderate grazing pressure with guard dogs exceeding legal limits, and (iii) no grazing and no tourism pressure. In total, 27 possible reintroduction sites were assessed, 8 in the Țarcu Mountains, 8 in the Ciucaș Mountains, and 11 in the Bucegi Mountains.

### 2.3. Statistical Analysis

Population dynamics were evaluated in the three main study areas where the alpine marmot is present: Retezat, Rodna, and Făgăraș; the population trends from 2004 to 2025 were analyzed. Compound Annual Growth Rate (CAGR) was calculated for the entire alpine marmot population to assess the overall trend and consistency of population change.

Principal component analysis (PCA) was performed to study the relationship between natural resources and the marmot population (marmot colonies, location, altitudes, plant traits, presence of water) [37]. Python 3.9.6. was used to perform the PCA. The categories of the altitudes were adapted to the altitudes where the marmot colonies have been observed and are the following: 1600–1800 m for low altitude; 1800–2000 m for medium altitude, and above 2000 m for high altitude.

Habitat Suitability Index (HSI) was constructed using a composite score derived from ecologically relevant variables that assess habitat quality and potential to identify the best colonies where marmots can be reintroduced. Although altitude, exposition, and slope were measured, they were not used in this analysis. This approach was considered more reliable and more easily reproducible in other areas with potential for alpine marmot colonization. The development of the HSI was based on four fundamental assumptions: (1) a higher number of active entrances indicates better habitat quality; (2) fewer abandoned entrances suggest better habitat; (3) larger territory sizes correlate with higher habitat quality; and (4) lower levels of anthropogenic influence enhance habitat suitability. To ensure a coherent analysis, all variables were initially standardized to accommodate differing measurement scales. Following standardization, a reverse scaling procedure was applied so that higher numerical values reflect lower habitat suitability. To simplify interpretation and support comparative analysis, HSI values for the 34 alpine marmot colony sites were reclassified into three suitability categories: Low (HSI < –0), Medium (0 ≤ HSI ≤ 0.35), and High (HSI > 0.351).

The Ecological Diagnostic Key for Marmot Habitats (EDK) was developed to assess the suitability for the three potential areas for alpine marmot reintroduction in the three possible mountains: Ciucaș, Bucegi, and Țarcu Mountains. The EDK was developed based on the concepts of Sustainable Population Threshold (SPT) [38] and Order 393/2002 [39,40]. As these mountains were not habituated by marmots, the HSI could not be applied, as there is no evidence about the active or abandoned entrance and territory sizes. The EDK was developed based on two groups for possible habituation factors: Abiotics, which included the altitude of the terrain (A) and the types of deposits (B) present in the area, and Biotics, which included the vegetation type (C) and the anthropogenic factor (D). The EDK was developed as a score-based diagnostic key, where each of the factors had a maximum of 50 points, while each subcategory had a maximum of 25 points. The Abiotic component included two criteria: altitude and surface type. Altitude was scored based on elevation bands, with the highest suitability (25 points) attributed to areas between 1700 and 1900 m, while lower or higher elevations received reduced scores. Surface type assessed the presence and configuration of rocky outcrops, with the maximum score assigned to well-developed rocky outcrops in glacial cirques, indicative of stable and protected terrain suitable for marmot burrowing. The Biotic components included vegetation composition and anthropogenic pressure. Vegetation was evaluated based on structure and productivity, where the most favorable habitats (25 points) consisted of mixes of high-productivity grasses with alpine clovers. Less suitable areas, dominated by mosses, lichens, or shrubs, received proportionally lower scores. Anthropogenic pressure was assessed by the intensity of grazing and the presence of livestock guardian dogs. Areas without grazing and tourism received the full 25 points, while regions with intensive grazing and excessive guarding dog presence scored lowest (0 points). Sites that obtained scores above 50 were considered highly suitable; those scoring between 31 and 50 points were classified as Medium suitability, while sites scoring below 30 points were considered to have Low suitability. This scoring framework allowed for the standardized evaluation of potential relocation sites, enabling comparison across regions and supporting evidence-based conservation planning.

## 3. Results

### 3.1. Alpine Marmot Population Growth in the Past Two Decades

**Figure 1:**
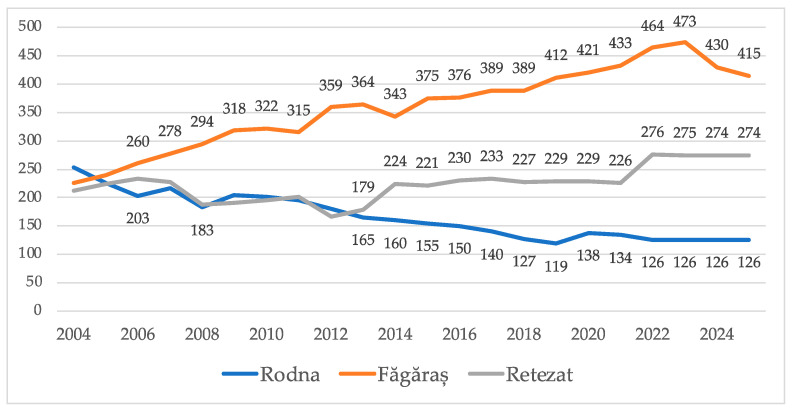
Temporal dynamics of alpine marmot populations in Făgăraș, Retezat, and Rodna Mountains between 2004 and 2025.

Between 2004 and 2025, the alpine marmot populations in the three core Romanian mountain regions, Făgăraș, Retezat, and Rodna, exhibited distinct trends in population dynamics (Figure 1), reaching 815 individuals in 2025. The Făgăraș Mountains showed a marked and consistent increase in population size, rising from 230 individuals in 2004 to a peak of 473 individuals in 2022, before a slight decline to 415 by 2025. In contrast, the Retezat Mountains experienced moderate growth. The population increased from 203 individuals in 2004 to 274 individuals by 2021, with subsequent stability in the following years. This pattern can suggest a saturation point in the region. The Rodna Mountains presented a declining trend, with the population dropping from 250 individuals in 2004 to 126 individuals by 2020, where it stabilized.

**Figure 2:**
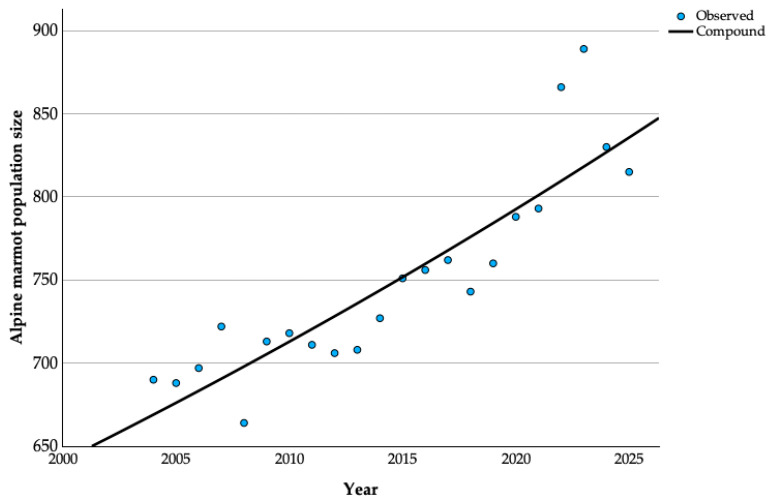
Compound Annual Growth Rate of the alpine marmot population in Romania (2004–2025).

The Compound Annual Growth Rate (CAGR) over the 22-year observation period (2004–2025) (Figure 2) was calculated at 1.1% per year, based on a statistically significant compound model (R^2^ = 0.802, *p* < 0.001) at a 95% confidence interval. During this period, the alpine marmot population in Romania exhibited a gradual but consistent upward trend, increasing by approximately 26% overall. The observed population values closely follow the fitted exponential curve, particularly after 2010, confirming a reliable long-term growth trajectory.

### 3.2. Relationship Between Natural Resources and the Marmot Population

**Figure 3:**
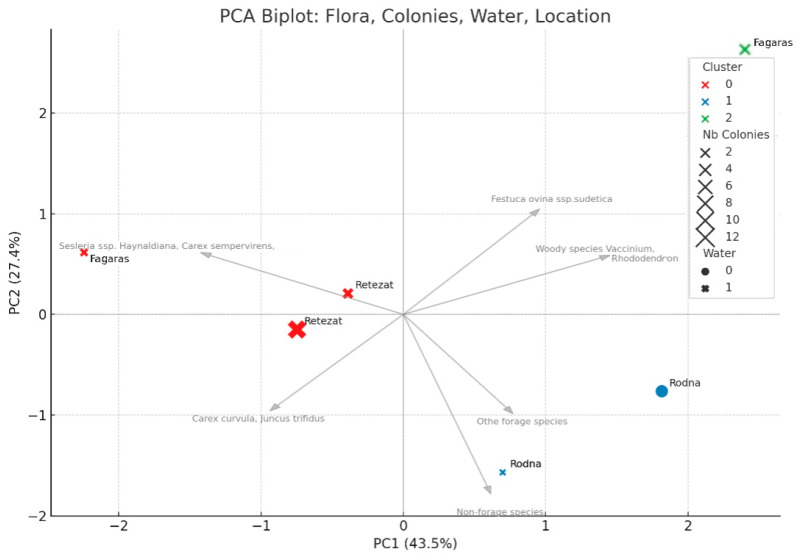
PCA of natural resources and number of colonies from the studied locations.

Several refuge burrows have been observed in the areas of the colonies from the studied locations showing that they can run different distances near their summer activity burrow, which is usually different than the hibernation burrow. The variation in the territory size of a colony is similar in the Retezat and Rodna Mountains but differs in the Făgăraș Mountains. In the latter, 70% of the colonies occupy less than 1 ha, while 30% range between 2 and 3 ha. The floristic relevés used to assess the vegetation are representative of the species’ habitat from all three mountain ranges, and we can only assume that the marmots’ diet includes the present floristic species. The *Festuca ovina* ssp. *sudetica* grasslands account for 28% of the total. This species has moderate nutritional value, along with *Agrostis impestris*. Grasslands dominated by *Carex curvula* and *Juncus trifidus* represent 25% of the total. Other forage species identified in these grasslands include *Festuca ovina* ssp. *sudetica*, *Agrostis rupestris*, *Poa alpina*, *Trifolium repens*, *Festuca violacea*, and *Ligusticum mutellina*. Among the non-forage species, the most frequently occurring are *Juncus trifidus*, *Luzula alpino-pilosa*, *Luzula sudetica*, *Festuca glacialis*, *Primula minima*, and *Campanula alpina*. Grasslands with *Sesleria rigida* ssp. and *Carex sempervirens* have a high palatability, up to 90%, but make up only 7% of the total. Within the 11% category of other forage species, the most commonly found are *Poa nemoralis*, *Trifolium alpestre*, *Festuca versicolor*, and *Festuca carpatica*. At the same time, the woody species occur in 14% of the total area, with *Vaccinium* and *Rhododendron* being the most frequent. The PCA biplot (Figure 3) summarizes the relationships among mountain groups, floral composition, water sources, and the number of marmot colonies, revealing ecological variability in a low-dimensional and interpretable form. The first two principal components together explain almost 71% of the total variance. The axes separate the sites primarily by altitude and, secondarily, by mountain range, with the Rodna Mountains clearly distinguished from the other groups. Marmot colonies are less numerous in areas dominated by woody species or other non-forage plants.

### 3.3. Habitat Suitability Index of the Already Colonized Regions

**Figure 4:**
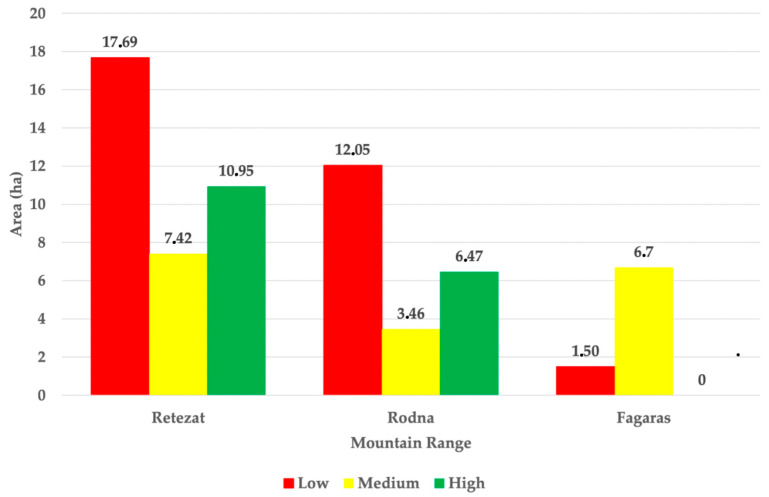
Habitat Suitability Index (HSI) values for alpine marmot colonies across the three mountain ranges in Romania.

Approximately 26.3% of colony sites fall into the High suitability class across the three mountain ranges, 20.6% into the Medium suitability class, and the remaining 47.2% into the Low suitability class (Figure 4). Among the 17 colonies located in the Retezat Mountains, the largest proportion, 30.4% (3 colonies), belongs to the High suitability class, covering a total area of 10.95 ha, followed by 4 colonies (20.6%) classified as Medium suitability, with a cumulative area of 7.42 ha, suggesting generally favorable conditions for alpine marmot populations in this region. In the Rodna Mountains, of the seven colonies analyzed, only one colony (6.47 ha) qualifies as High suitability, while two colonies, with a combined area of 3.46 ha, fall into the Medium suitability class. In contrast, none of the 10 colonies assessed in the Făgăraș Mountains meet the criteria for High suitability; the majority, 81.7%, belong to the Medium suitability class, covering 6.7 ha, while the remaining 1.5 ha are categorized as Low suitability.

### 3.4. Ecological Diagnostic Key of the Possible Introduction Sites of the Alpine Marmot

**Figure 5:**
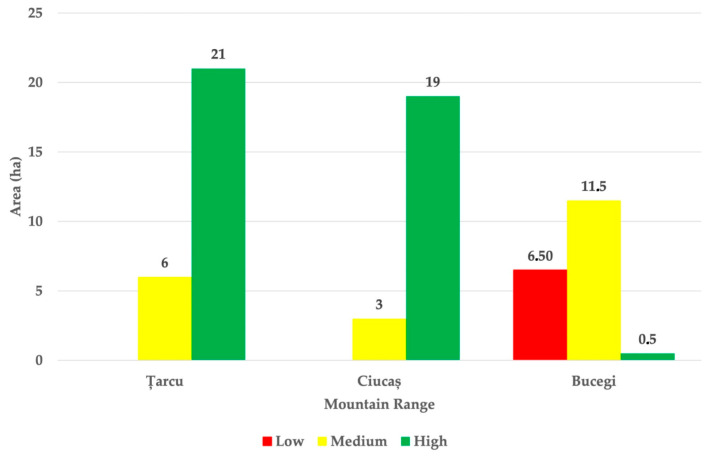
Suitability classification of potential alpine marmot reintroduction sites based on EDK scores in Țarcu, Ciucaș, and Bucegi Mountains.

Out of the 27 possible reintroduction sites, 40.5 ha were classified as highly suitable for alpine marmot reintroduction, 20.5 ha as moderately suitable, and the remaining 6.5 ha as Low suitability (Figure 5) based on the results of the Ecological Diagnostic Key (EDK). While both the Țarcu and Ciucaș Mountains included areas with Medium and High suitability, the Bucegi Mountains contained the largest proportion of unsuitable areas, indicating zones where reintroduction should be avoided. Based on these estimations, an area of 61 ha from all three mountain ranges is theoretically suitable for marmot reintroduction.

## 4. Discussion

This study confirms that the alpine marmot population in Romania has remained stable over the past two decades, showing a slightly increasing growth trend that continues the pattern established since the reintroduction efforts in the Retezat, Rodna, and Făgăraș Mountains. Similar growth trends following reintroduction have been recorded in the Dolomiti Bellunesi National Park, where the population surpassed the number of released individuals within just two years [41]. In the Apennine ridge, reintroduced marmots have successfully expanded their range, now occupying over 700 km^2^ [42]. In the Pyrenees, the introduction of approximately 500 individuals between 1948 and 1988 led to a rapid population expansion, with the species currently occupying an area of 8200 km^2^, demonstrating one of the highest post-reintroduction spread rates documented in Europe [20]. These studies confirm that marmot reintroduction is highly feasible and can lead to rapid population growth and range expansion when suitable habitat conditions are met. Moreover, it is proven that capturing of the relocated individuals is unlikely to intensify the effects of human disturbances [43].

The PCA highlights differences between the three studied mountain groups. The Rodna Mountains are apparently different than the other two locations, having the highest percentages of non-forage plants and other plants with low forage value. Although further studies are required to confirm the relationship, we hypothesize that the presence of water sources plays a critical role in the stability of marmot colonies. We noticed in Rodna several abandoned burrows all grouped nearby, indicating that a colony moved from the place with similar landscape characteristics as the others, but lacking permanent water. Similar studies support this idea, showing that the distribution of *Marmota marmota* is influenced not only by vegetation and topography, but also by proximity of permanent water sources [44]. On the other hand, studies support the idea that hoary marmots prefer plants that retain water, and having water sources nearby the burrows is not so vital compared to other landscape features as slope, aspect, and presence of shrub vegetation [45]. This study used PCA to better understand the ecological conditions of current marmot habitats and to assess whether similar environments could support future reintroduction efforts [46,47]. The analysis was further deepened by HSI evaluation which provided a holistic view of the habitat conditions of the studied marmot colonies. While PCA allowed identification of assumed key environmental features, the HSI integrated multiple habitat variables aligned to the ecological requirements of marmots.

The Habitat Suitability Index shows that the most robust marmot colonies, those in the Retezat, Rodna, and Făgăraș Mountains, occupy about 17 ha of high-quality habitat and can safely serve as donor sites. The translocation strategy should consider the donor population as well. This should be a robust, stable, or increasing population, rather than a declining one. Also, fewer relocated individuals than the carrying capacity should be considered to ensure sustainability of the source population and to be cost effective and efficient, and to allow them time to accommodate to the new areas [48]. With the national population now estimated at roughly 815 individuals in 2025, removing no more than five percent (around 40 animals) would not jeopardize local demography, provided the removals are spread proportionately among ranges [48,49,50]. Rodna, holding nearly 400 marmots, could contribute 18–20 mostly juvenile or sub-adult individuals; Retezat, with about 300 animals could supply 12–15; and Făgăraș, the smallest population at roughly 115 individuals, could donate up to 6 without measurable demographic risk. Translocated groups should number fifteen to twenty marmots per release site, with balanced sex ratios and a bias toward yearlings and sub-adults to maximize social cohesion and post-release survival [49]. Because all Romanian colonies trace back to the same 1970s stocks from Austria and France [25], genetic diversification is advisable [51]. Introducing 30–40 additional marmots from genetically stable populations, such as those from the Alpine arc from Gran Paradiso to Mercantour National [52], would inject new alleles and reduce inbreeding risk. Removing this modest number would represent only a minute fraction of the donor populations, making the impact on those source areas ecologically negligible.

New colonies should be established first in the highest-suitability zones identified by the Ecological Diagnostic Key (EDK) in the Țarcu and Ciucaș Mountains; the Bucegi range, by contrast, remains heavily impacted by tourism and grazing and should be considered only after targeted mitigation. While some studies highlight that tourism can negatively affect marmot population [53,54], in the areas we observed (Retezat, Rodna and Făgăraș), the impact seemed minimal. The marmots’ behavior was monitored, and they did not appear to be strongly disturbed by the presence of tourists. This was proven by another study as well, concluding that the tolerance of the yellow-bellied marmot colony for human disturbances can increase over time [55]. Instead, their reactions were more closely tied to the type of potential threat (whether it came from the air or on foot) and they relied mainly on nearby burrow options rather than responding specifically to people. However, anthropogenic factors must be considered when planning such measures because mitigating their negative effects is nearly impossible; current livestock management practices rely on guard dogs to protect herds from brown bears and gray wolves [56,57]. A phased reintroduction strategy, two release waves of roughly 20 animals each, spaced two years apart, will allow managers to evaluate early survival and refine release protocols before the second introduction. Because the six Romanian mountain ranges are isolated and share no natural gene flow, alpine marmot management must follow a cautious, genetics-informed approach to safeguard long-term diversity. Together, well-distributed removals from Romanian source colonies and a limited infusion of genetically diverse founders from outside sources offer a pragmatic framework for expanding the species’ range, enhancing genetic diversity and sustaining the positive population trajectory recorded over the past two decades. To avoid any genetics-related issues, a gene-flow infusions should also be considered for the established alpine marmot populations in the Retezat, Rodna, and Făgăraș Mountains. However, not only source population should be considered, but also the release protocols.

The reintroduction protocol should rely on soft-release methods that combine a brief acclimation period with purpose-built shelters [40]. For alpine marmots, these shelters should be artificial burrows approximately 15–20 cm in diameter and 2–4 m in length, positioned on south- to southwest-facing slopes to maximize solar gain and drainage. The terminal chamber should be lined with hay, and the entrance should be lightly backfilled with loose soil so that the animals must reopen it; this digging activity encourages site fidelity and reduces immediate long-distance dispersal [41]. Artificial burrows are best deployed in clusters of three to five, spaced 50–100 m apart. In addition, electric fencing should be installed around the release area to deter predators and livestock-guardian dogs, thereby increasing post-release survival. Although alpine marmots generally do not provoke direct conflict with humans, increased tourism and the presence of off-leash dogs [58] in reintroduction zones could present challenges. Management plans should include public awareness campaigns and seasonal access regulations, as well as annual survival monitoring of the relocated individuals, with regular visual observations to assess burrow use, foraging activity, social interactions, predator response, presence of juveniles, mapping of activity areas, and preferred foraging locations [37,49].

While this study provides valuable insights into the population trends and habitat suitability of alpine marmots in Romania, several important aspects remain unaddressed. First, a detailed analysis of the genetic structure of Romanian alpine marmot populations is necessary to determine whether genetic supplementation is required and to assess the potential risks of inbreeding or genetic drift. Second, although habitat suitability was evaluated using well-measured ecological variables, the analysis did not include fine-scale factors such as burrow microclimate conditions or predation pressure. These elements can significantly influence colony persistence and reproductive success and should be integrated into future research. Additionally, while most of the studied areas fall within protected zones, the influence of wildlife management practices, hunting pressure, and enforcement of conservation regulations should be considered, as these factors directly affect the stability and safety of existing populations. Moreover, although ecological data were used to identify suitable reintroduction sites, no formal assessment was made regarding the potential impact of climate change [59]. At the moment, we can only assume that snow cover is a major cause of mortality for the marmot population in Romania. Because Romanian colonies face one of Europe’s highest densities of brown bear, wolf and lynx, small reintroduced groups may become perpetual sink populations unless releases are large enough to cover predation losses. High-use hiking corridors, particularly in the Bucegi and Făgăraș massifs, create a near-constant human presence; experimental work in Switzerland shows even low-level tourist traffic triggers flight responses and reduces foraging time [60]. In the context of climate change, snow cover may persist for too many months, causing population collapse, or may become too thin to provide proper insulation, compromising the temperature conditions necessary for the species’ survival. Future studies should evaluate how shifts in temperature and snow cover may affect hibernation cycles, food availability, and overall habitat suitability for the species. Lastly, even though the alpine marmot is a low-conflict species, the attitudes and perceptions of local communities were not explored.

## 5. Conclusions

This study provides a framework for guiding the future conservation and range expansion of the alpine marmot in Romania. Beyond confirming population stability, it highlights the need for a more integrated approach that combines ecological, demographic, and genetic data with practical field methods. Effective conservation of this species will depend on refining reintroduction protocols, ensuring genetic viability, and minimizing anthropogenic pressures in both current and prospective habitats. As pastoral practices decline in popularity and the current marmot population remains stable, efforts should now focus on the reintroduction of the species into new suitable areas within Romania.

## Figures and Tables

**Table 1:** Description of variables collected for the Habitat Suitability Index.

Variable	Code	Type	Min.	Max.	Mean	Type
Altitude	ALT	Numerical	1600	2324	2006	Numerical
Exposition	EXP	Categorical (8 levels)				Ordinal
Slope	SL	Numerical	10	40	25.32	Numerical
Territory size	TS	Numerical	0.3	6.47	1.95	Numerical
Active entrance	AE	Numerical	0	50	8.18	Numerical
Abandoned entrance	ABE	Numerical	0	30	4.56	Numerical
Anthropic factor	AF	Categorical (3 levels)				Ordinal

## Data Availability

The data will be made available upon request.
